# FAILURE TO RESCUE AFTER GASTRECTOMY: A NEW INDICATOR OF SURGICAL QUALITY

**DOI:** 10.1590/0102-672020230056e1774

**Published:** 2023-11-13

**Authors:** Stefany HONG, Marina Alessandra PEREIRA, André Roncon DIAS, Ulysses RIBEIRO, Luiz Augusto Carneiro D’ALBUQUERQUE, Marcus Fernando Kodama Pertille RAMOS

**Affiliations:** 1 Universidade de São Paulo, University Hospital, Faculty of Medicine, Department of Gastroenterology, São Paulo (SP), Brazil.

**Keywords:** Stomach Neoplasms, Gastrectomy, Postoperative Complications, Indicators of Quality of Life, Neoplasias Gástricas, Gastrectomia, Complicações Pós-Operatórias, Indicadores de Qualidade de Vida

## Abstract

**BACKGROUND:**

The main treatment modality for gastric cancer is surgical resection with lymphadenectomy. Despite advances in perioperative care, major surgical complications can occur in up to 20% of cases. To determine the quality of surgical care employed, a new indicator called failure to rescue (FTR) was proposed, which assesses the percentage of patients who die after complications occur.

**AIMS:**

To assess the rate of FTR after gastrectomy and factors associated with its occurrence.

**METHODS:**

Patients with gastric cancer who underwent gastrectomy with curative intent were retrospectively evaluated. According to the occurrence of postoperative complications, patients were divided into FTR group (grade V complications) and rescued group (grade III/IV complications).

**RESULTS:**

Among the 731 patients, 114 had major complications. Of these patients, 76 (66.7%) were successfully treated for the complication (rescued group), while 38 (33.3%) died (FTR group). Patients in the FTR group were older (p=0.008; p<0.05), had lower levels of hemoglobin (p=0.021; p<0.05) and albumin (p=0.002; p<0.05), and a higher frequency of ASA III/IV (p=0.033; p<0.05). There were no differences between the groups regarding surgical and pathological characteristics. Clinical complications had a higher mortality rate (40.0% vs 30.4%), with pulmonary complications (50.2%) and infections (46.2%) being the most lethal. Patients with major complications grade III/IV had worse survival than those without complications.

**CONCLUSIONS:**

The FTR rate was 33.3%. Advanced age, worse performance, and nutritional parameters were associated with FTR.

## RESUMO

**RACIONAL:**

A principal modalidade de tratamento do câncer gástrico (CG) é a ressecção cirúrgica associada a linfadenectomia. Apesar dos avanços no cuidado peri-operatório, as complicações cirúrgicas maiores podem ocorrer em até 20% dos casos. Para determinar a qualidade do atendimento cirúrgico empregado, foi proposto um novo indicador denominado *failure to rescue* (FTR), que avalia a porcentagem dos pacientes que vão a óbito após a ocorrência de complicações.

**OBJETIVOS:**

Avaliar a taxa de FTR após gastrectomia e fatores associados com a sua ocorrência.

**MÉTODOS:**

Foram avaliados retrospectivamente os pacientes com CG submetidos à gastrectomia com intenção curativa. De acordo com a ocorrência de complicações pós-operatórias, os pacientes foram divididos em grupo FTR (complicação grau V) e grupo resgatado (complicações grau 3-4).

**RESULTADOS:**

Entre os 731 pacientes, 114 tiveram maiores complicações. Desses pacientes, 76 casos (66,7%) obtiveram sucesso no tratamento da complicação (grupo resgatados), enquanto 38 casos (33,3%) foram a óbito (grupo FTR). Os pacientes do grupo FTR apresentaram idade mais avançada (p=0,008, p<0,05), menores níveis de hemoglobina (p=0,021, p<0,05) e de albumina (p=0,002, p<0,05), e maior frequência de ASA III/IV (p=0,033, p<0,05). Não houve diferença entre os grupos quanto às características cirúrgicas e patológicas. As complicações clínicas apresentaram maior taxa de mortalidade (40.0% *vs* 30,4%), sendo complicações pulmonares (50,2%) e infecções (46,2%) as mais letais. Pacientes com complicações maiores grau III-IV tiveram pior sobrevida comparado aqueles sem complicações.

**CONCLUSÕES:**

A taxa de *FTR* foi de 33,3%. Idade avançada, pior performance, e parâmetros nutricionais associam-se ao FTR.

## INTRODUCTION

Gastric cancer (GC) is an important disease and still the fifth most common cancer worldwide. In Brazil, in the year 2023, it is estimated the occurrence of 21,480 new cases of GC^12^. Predisposing factors, such as smoking and alcoholism, as well as the commonly associated malnutrition as the disease progresses, could make patients more fragile^17^.

The main modality of treatment for GC is resection of the tumor with free margins and removal of regional lymph nodes^2,8^. Unfortunately, to achieve this objective, the procedure can have considerable morbidity and mortality. We have experienced improvements in treating these patients through the standardization and centralization of treatment in reference cancer centers, the greater use of minimally invasive (MI) surgery, and the association with systemic chemotherapy^19^. Currently, the frequency of postoperative morbidity and major complications in Western centers is around 20%^3,6^.

Traditionally, to assess the quality of surgical treatment of GC, the most used indicator is the incidence of postoperative complications. There is no doubt that this is a relevant indicator; however, no matter how careful and expert the service is provided, a certain number of complications is still expected. Considering that this occurrence is inevitable, Silber et al. first described the term failure to rescue (FTR), defined as the death rate among patients who presented postoperative complications after myocardial revascularization surgery^18^. The rationale behind this indicator is: If the occurrence of complications is inevitable, what is our service’s ability to manage them successfully?

This concept has gained prominence in recent decades as it has proven to be a strong quality indicator that reflects not only the surgeon’s performance but also the entire multidisciplinary support network that the patient receives during his treatment. In the current literature, the FTR in patients undergoing gastrectomy varies around 25% in European centers (Germany and Netherlands), 28% in Australia and New Zealand, and as low as 4.53% in South Korea^5,6,9,11,20,21^.

In our institution dedicated to cancer treatment, we constantly seek improvements in perioperative care, nutritional support, and training of the multidisciplinary team. In recent years, several publications of our results have already confirmed the quality of surgical care in complications^7,10,16^. However, the analysis of our FTR has not yet been carried out to determine and identify its main causes to guide new actions to improve our results.

This study aimed to assess the rate of FTR after gastrectomy and the factors associated with its occurrence.

## METHODS

A retrospective review of all patients with GC who underwent surgical treatment from 2009 to 2022 was performed. Patients with gastric tumors of the histological adenocarcinoma type who were subjected to curative intent gastrectomy with lymphadenectomy (D1 or D2) were included. Patients who underwent diagnostic surgery, conversion surgery, and gastric remnant tumors were excluded.

Postoperative complications were graded according to the Clavien-Dindo classification, in which grades III to V were defined as major postoperative complications. Patients who had major complications and died during hospitalization were considered in the FTR group. In a secondary analysis, patients were divided into two groups, according to the year of treatment (2009–2015 and 2016–2022), to assess the evolution of the indicator.

Preoperative clinical variables evaluated included sex, age, body mass index (BMI) (kg/m^2^), hemoglobin (g/dL) and albumin (g/dL) levels, neutrophil-lymphocyte ratio (NLR), Charlson-Deyo comorbidity index (CCI) and American Society of Anesthesiologists (ASA) physical status classification. Preoperative chemotherapy was indicated in cases with clinical staging T3/T4 and/or positive lymph nodes (LN).

Total or distal gastrectomy was performed according to the location and size of the tumor to obtain an R0 resection. The extension of the lymph node dissection (D1 or D2), as well as the surgical access (open or MI), were defined by the attending surgeon of the case. All procedures were performed according to the guidelines of the Japanese Gastric Cancer Association (JGCA) and the Brazilian Gastric Cancer Association, by a highly experienced surgical team^2,8^. Late outcomes evaluated included recurrence and death.

Patients were followed up at outpatient clinical appointments according to a standard protocol, with consultations every three months in the first year, every six months in the second and third years, and thereafter, once a year.

### Statistical analysis

Descriptive statistics were presented as frequencies for categorical variables, and mean with standard deviation (±SD) or median with interquartile range (IQR) for continuous variables. Categorical variables were compared using Pearson’s chi-square test, while continuous variables used Student’s *t*-test. Variables associated with FTR were analyzed by binary logistic regression, and odds ratios (OR) with a 95% confidence interval (95%CI) were calculated. Variables with p-value (p)<0.2 in the univariate analysis were included in the multivariate model.

Survival was estimated applying the Kaplan-Meier method, and differences between the curves were assessed by the log-rank test. Disease-free survival (DFS) was calculated from surgery to recurrence or death from any cause, and overall survival (OS) from surgical resection to death. All statistical tests were two-sided and p<0.05 were considered significant. Statistical analyses were performed using Statistical Package for Social Sciences (SPSS) software, version 20 (Chicago, IL). The study was approved by the Ethics Committee of the institution (CAAE:68661023.8.0000).

## RESULTS

**Figure 1:**
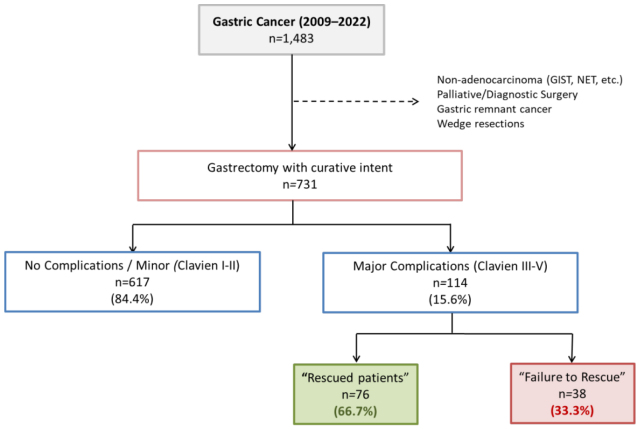
Study flowchart.

GIST: gastrointestinal tumor; NET: neuroendocrine tumor.

From 2009 to 2022, 1,483 patients underwent surgery for GC at our institution. Among them, 731 patients with gastric adenocarcinoma who were submitted to surgery with curative intent were included (Figure 1). Major complications (Clavien-Dindo=3) occurred in 114 patients (15.6%), and among those, 38 died during hospitalization, defining an FTR rate of 33.3%.

The clinical characteristics of patients in both groups are shown in Table 1. Patients in the FTR group were older (p=0.008; p<0.05), had lower levels of hemoglobin (p=0.021; p<0.05) and albumin (p=0.002; p<0.05), and had worse functional status according to the ASA classification (p=0.033; p<0.05). No significant difference was observed between the groups concerning preoperative chemotherapy, surgical access, and type of gastrectomy.

**Table 1:** Clinical and surgical characteristics of rescued and failure to rescue patients.

Variables	Rescued patients	Failure to rescue	p-value

	n=76 (%)	n=38 (%)	
Sex			
Female	26 (34.2)	11 (28.9)	0.527
Male	50 (65.8)	27 (71.1)	
Age (years)			
Mean (±SD)	64.9 (±10.1)	70.3 (±10)	0.008
BMI (kg/m²)			
Mean (±SD)	24.1 (±4.9)	25.6 (±6.9)	0.253
Hemoglobin (g/dL)			
Mean (±SD)	12.5 (±2.1)	11.5 (±2.4)	0.021
Albumin (g/dL)			
Mean (±SD)	4.0 (±0.6)	3.6 (±0.6)	0.002
Neutrophil-to-lymphocyte ratio			
Mean (±SD)	2.81 (±2.62)	3.08 (±2.08)	0.584
Charlson-Deyo Comorbidity Index (CCI)			
CCI=0	43 (56.6)	16 (42.1)	0.145
CCI=1	33 (43.4)	22 (57.9)	
ASA			
I/II	48 (63.2)	16 (42.1)	0.033
III/IV	28 (36.8)	22 (57.9)	
Preoperative chemotherapy			
No	57 (75.0)	31 (81.6)	0.430
Yes	19 (25.0)	7 (18.4)	
Surgical technique			
Open	58 (76.3)	34 (89.5)	0.093
Minimally invasive	18 (23.7)	4 (10.5)	
Extent of gastrectomy			
Subtotal	43 (56.6)	23 (60.5)	0.687
Total	33 (43.4)	15 (39.5)	

SD: standard deviation; BMI: body mass index; ASA: American Society of Anesthesiologists classification.

Pathological characteristics are demonstrated in Table 2. Most patients had intestinal subtype adenocarcinoma and advanced stage II/III tumors.

**Table 2:** Pathological characteristics of rescued and failure to rescue patients.

Variables	Rescued patients	Failure to rescue	p-value

	n=76 (%)	n=38 (%)	
Size			
Mean (±SD)	4.9 (±3.6)	4.8 (±3.3)	0.880
Histological subtype			
Intestinal	43 (56.6)	27(71.1)	0.135
Diffuse	33 (43.4)	11 (28.9)	
Differentiation			
G1/G2	39 (51.3)	25 (65.8)	0.142
G3	37 (48.7)	13 (34.2)	
pT			
pT1/T2	35 (46.1)	14 (36.8)	0.349
pT3/T4	41 (53.9)	24 (63.2)	
pN			
pN0	30 (39.5)	17 (44.7)	0.590
pN+	46 (60.5)	21 (55.3)	
pTNM			
I	24 (31.6)	11 (28.9)	0.923
II	17 (22.4)	8 (21.1)	
III	35 (46.1)	19 (50.0)	

SD: standard deviation; G:grade; pT: primary tumor; pN: lymph node metastasis; pTNM: pathological tumor-node-metastasis.

**Figure 2:**
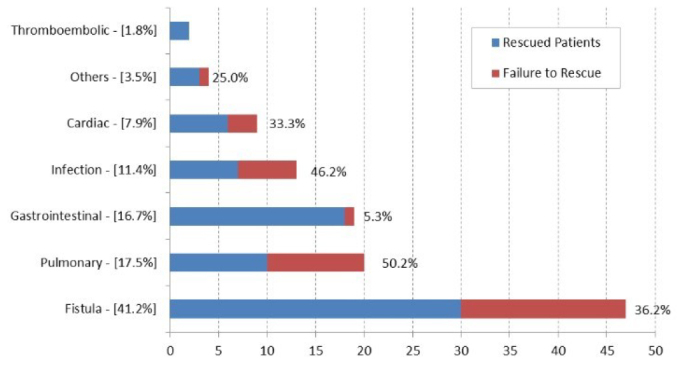
Incidence of major complications and rate of failure to rescue.

Figure 2 describes the incidence of FTR by type of major complications after gastrectomy. Fistula was the most common, with an incidence of 41.2% and a FTR rate of 36.2%. The second most common was pulmonary complication (17.5%); however, it had the highest rate of FTR (50.2%). The following complications with the highest rate of FTR were infection (46.2%) and cardiac (33.3%).

**Figure 3:**
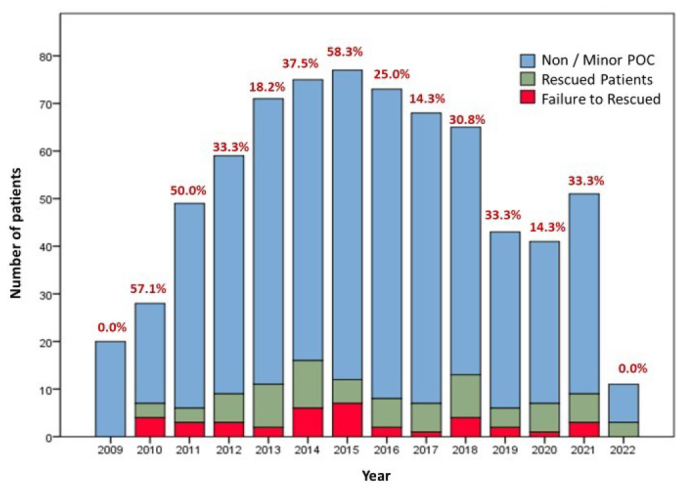
Histogram demonstrating the number of patients and rate of failure to rescue during the period of the study.

The evolution of the indicator during the study period is shown in Figure 3. Further, we divided the analysis into two periods. In the first period, comprising the interval from 2009 to 2015, the rate was 49.0%. However, from 2016 to 2022, we noticed a decrease in mortality, with a rate of 24.5% (p=0.063).

POC: postoperative complications.

The univariate analysis of variables related to the occurrence of FTR demonstrated that age ≥65 years, ASA III/IV, low hemoglobin and albumin levels, and surgery performed from 2009 to 2015 were associated. After multivariate analysis, only albumin levels ≤3.4 g/dL (OR=2.7; 95%CI 1.04–12.51; p=0.044) and surgery performed from 2009 to 2015 (OR=3.61; 95%CI 1.22–10.74; p=0.021) maintained the association (Table 3).

**Table 3:** Univariate and multivariate analysis of variables associated with failure to rescue.

Risk for failure to rescue	Univariate			Multivariate*		

Variables	OR	95%CI	p-value	OR	95%CI	p-value
Male (vs female)	1.28	0.55–2.97	0.572			
Age ≥65 years (vs <65 years)	2.59	1.13–5.95	0.025	2.04	0.75–5.51	0.162
ASA III/IV (vs ASA I/II)	2.36	1.07–5.21	0.035	2.46	0.75–8.07	0.137
CCI=1 (vs CCI=0)	1.79	0.81–3.94	0.147	1.00	0.34–3.01	0.994
Hemoglobin <11 g/dL (vs ≥11 g/dL)	4.05	1.77–9.27	0.001	2.70	0.95–7.70	0.063
Albumin <3.4 g/dL (vs ≥3.4 g/dL)	4.57	1.66–12.56	0.003	3.60	1.04–12.51	0.044
2009–2015 (vs 2016–2022)	2.14	0.95–4.79	0.065	3.61	1.22–10.72	0.021
Open (vs MI)	2.64	0.82–8.44	0.102	0.78	0.19–3.24	0.731
Total gastrectomy (vs subtotal)	0.85	0.39–1.88	0.688			
pT3/T4 (vs T1/T2)	1.46	0.66–3.25	0.350			
pN+ (vs pN0)	0.81	0.37–1.77	0.591			

* Variables with p<0.2 in the univariate analyses were included in the multivariate. CCI: Charlson-Deyo Comorbidity Index; MI: minimally invasive; ASA: American Society of Anesthesiologists classification; pT: primary tumor; pN: lymph node metastasis.

### Survival analysis

**Figure 4:**
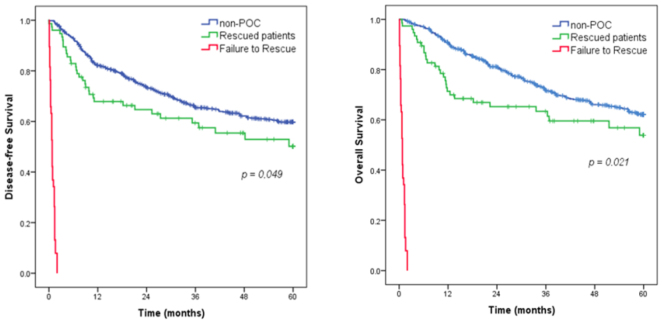
Survival curves of overall survival and disease-free survival according to the groups failure to rescue, rescued and non-postoperative complications groups.

Patients in the FTR group died in less than 30 days in 62.3% of cases and none of the others reached 90-day survival. Regarding the remaining patients, the rescued group had lower DFS and OS than the group of patients who had no complications or had minor complications (p=0.049 and p=0.021, respectively), as demonstrated in Figure 4.

POC: postoperative complications.

## DISCUSSION

In the present study, carried out in a single reference center in Brazil, the overall rate of FTR was 33.3% and was associated with age, nutritional status, and patient performance. Nowadays, cancer treatment is being centralized in reference centers in an attempt to improve surgical outcomes. Despite the morbidity inherent to the disease itself, it is well known that the uneventful postoperative evolution of the patient depends on the action of the entire multidisciplinary team that assists him. Early recognition of complications, whether surgical or clinical, and the adoption of adequate measures of treatment will directly impact the success of the patient’s rescue^14^.

From the introduction of the FTR concept by Silber et al.^18^, initially, for postoperative patients of myocardial surgery, until today, the use of this new indicator has been expanded. Researchers have been trying to identify the factors associated with the FTR, such as clinical characteristics of patients, surgical techniques employed^20^, socioeconomic factors, structure and level of hospital care^8^, and technical training of the multidisciplinary team that provides support to the patient throughout the hospitalization period.

Among the possible characteristics associated with FTR, we identified the older age and higher ASA category. Both reflect the high fragility of this group of patients with less physiological reserve to face a critical complication. It is common to find GC patients with poor clinical status due to a late diagnosis in undeveloped countries like Brazil. Therefore, performing an early diagnosis of GC is one effective measure to decrease morbidity and mortality. Low levels of hemoglobin and albumin are considered indicators of malnutrition, and both were also associated with FTR, once again demonstrating the fragility of these patients. If age is immutable and, in most cases, the ASA score is as well, preoperative nutritional support is an action with proven effectiveness and, consequently, has a potential impact on improving the FTR rate.

Another hypothesis would be that more advanced tumors could be associated with a higher occurrence of FTR. No significant data were found to validate this association, emphasizing the importance of the factors described above. In a recent study performed at our institution, it was demonstrated that the use of preoperative chemotherapy did not increase the incidence of complications^7^. We found a similar result and the rate of FTR was not associated with preoperative chemotherapy. It is even believed that this procedure may have a protective effect on the group of patients who have complications and are rescued since they will hardly be able to perform adjuvant treatment. The deleterious effect of complications on survival was verified even in resected patients, but due to the small number of cases, we could not analyze whether preoperative chemotherapy had a protective effect in our series^4,13^.^.^

Similarly, MI surgery has been proven to be a safe and effective technique, without demonstrating inferiority to the open technique, which continues to be the most common surgical approach in our service and worldwide^1^. It has been suggested that due to its minor inflammatory response, MI would have a better FTR rate. We did not find this association, but the small number of MI cases may have underpowered the analysis.

Previous studies have shown higher occurrence of complications after total gastrectomy than subtotal, but we found no difference in FTR. Interestingly, fistulas were the most common major complication in the study. It has been previously described that the duodenal stump fistula has the highest lethality in our service, affecting both subtotal and total resections similarly. On the other hand, the fistula of the esophagojejunal anastomosis is exclusive to patients submitted to total resection. For many years, it was the most feared by surgeons; however, recent advances, such as the use of prostheses and endoscopic vacuum therapy, have greatly reduced its lethality^10,16^.

We noticed that in our service, clinical complications had higher rates of FTR, with pulmonary complications having a 50.2% mortality rate and infection having 46.2%. Our results were similar to the Korean case series, which showed 50.0% mortality due to acute respiratory distress, 31.8% to coronary disease, followed by 17.2% to bleeding, and only 8.7% to fistulas^15^. In Australia, clinical complications also corresponded to the highest rates of FTR, with respiratory complications being responsible for 33.1% and cardiac for 11.5%^2^ of the cases. On the other hand, in European centers, we noticed a predominance of higher FTR due to surgical causes. In the series from the Netherlands, the main rates of FTR were due to bleeding (36%), dehiscence of the anastomosis (26%), cardiac (22%) and pulmonary complications (15%)^3^. In Germany, over the years studied, there was a reduction in FTR due to clinical causes from 71.2% to 18.2%, while surgical causes increased from 28.8% to 81.8%^2^.

Perioperative care, involving nutritional support, nursing staff, intensive care unit, and well-defined institutional protocols for adequate management of postoperative intercurrences, is the key to early identification and long-term success, with reduced morbidity and mortality.

Another objective of the study was to perform a temporal evaluation, considering data of the last 14 years from our institution. Comparing the intervals, we note that from 2009 to 2015 we had higher FTR rates. We attribute the constant changes that our service has undergone, both from the point of view of implementing innovations and the increase in the implementation of assistance protocols such as the Enhanced Recovery after Surgery (ERAS)^14^. It is important to maintain a constant search to improve the training and appreciation of the various members of our multidisciplinary team, formed by the areas of nursing, nutrition, psychology, social assistance, and other medical specialties, thus composing an equipotent support network for our patients.

A limitation of the present study is the absence of data regarding the performance of the multidisciplinary team participating in the patient’s assistance. Due to the retrospective nature of the study and its long period, we could not assess precise information in this relation. Respiratory therapy sessions, nutritional therapy employed, and intensive care could not be evaluated. Recently, the ERAS protocol^14^ became part of the institutional routine, facilitating and allowing the collection of these data for future analysis. As positive points, we were able to analyze a considerable number of cases in a Western center with extensive expertise. The indicator’s temporal improvement trend was a stimulating result for us, and we believe that, with the adoption of new interventions, we will be able to improve it. If we may have reached the limit of preventing the occurrence of surgical complications, their adequate treatment still has a margin for improvement.

## CONCLUSIONS

Patients who underwent GC treatment in our institution presented an FTR rate of 33.3% associated with advanced age, malnutrition, and poor performance. In the most recent period, the indicator showed improvement, but even patients who were rescued had worse long-term survival.
